# Unravelling the impact of linear energy transfer on micronuclei induction from proton and photon irradiation

**DOI:** 10.1038/s41598-025-09763-9

**Published:** 2025-07-06

**Authors:** Charlotte J. Heaven, John-William Warmenhoven, Amy L. Chadwick, Elham Santina, Jamie Honeychurch, Christine K. Schmidt, Karen J. Kirkby, Michael J. Merchant, Nicholas T. Henthorn

**Affiliations:** 1 Manchester Academic Health Science Centre, The Christie NHS Foundation Trust, Wilmslow Road, Manchester, M20 4BX UK; 2 Division of Cancer Sciences, Faculty of Biology, Medicine and Health, School of Medical Sciences, The University of Manchester, Oxford Road, Manchester, M13 9PL UK

**Keywords:** Micronuclei, cytochalasin-B, Radiotherapy, Proton therapy, DNA damage, Radiotherapy, Tumour immunology, DNA damage response

## Abstract

Micronucleus (MN) formation has a strong link to radiation damage and is a common bio-dosimeter for acute radiation exposures. The percentage of cells containing MN (PCMN) has a strong relationship with dose, however variation between previous studies has made understanding the effect of linear energy transfer (LET) difficult. This study investigated the PCMN of seven cell lines in response to photon and proton irradiation at two different LETs (0.6 keV/ µm and 6.5 keV/ µm). MN production was scored via the cytokinesis block micronuclei assay. A linear relationship between dose and PCMN was noted for all cell lines and radiation types, with a large variability in the MN yield between cell lines. This dose-dependent increase in PCMN was independent of LET, with most cell lines showing similar responses to the radiation qualities. Overall, this data proposes a more complex relationship between dose, MN and LET.

## Introduction

Micronuclei (MN) formation is routinely used as a bio-dosimeter for acute radiation exposures^1^ and has historically been used as a measure of DNA damage in cells^2,3^. MN can be formed from whole centromeres lost during cell division due to damage to the mitotic apparatus or can form from acentric fragments caused by DNA damage^4^. Ionising radiation induces cellular DNA damage, with the most lethal being the creation of double strand breaks (DSBs). These breaks can repair correctly; incorrectly; or can remain as residual damage, where incorrect or residual repair may lead to chromosomal aberrations. Following cell division, fragments of DNA not attached to the centromere cannot be taken into daughter cells and remain as exposed DNA in the cytoplasm. As the new daughter nuclei form, the lost fragments of DNA may gather leftover nuclear envelope proteins and form a smaller (micro) nucleus within the new cell. These MN are fragile in comparison to the main nucleus and are prone to rupture^3,5^. Not all DNA fragments are drawn into MN and not all MN are prone to rupture^6^. Several recent studies have explored the properties of MN with particular emphasis on differences in the DNA damage and micro-nuclear DNA sequence and structure (3, 5).

Interest in MN has recently grown due to their association with the immune response to radiation through the cyclic GMP–AMP synthase/ stimulator of interferon genes (cGAS /STING) pathway^7^. The cGAS/STING pathway responds to cytosolic DNA and triggers innate and adaptive immune responses^8^. It has been suggested to be a major contributor to abscopal effects from ionising radiation and its activation is the focus of many studies aiming to hijack the pathway for use in cancer treatment^9^. MN are closely associated with cGAS/STING and studies have suggested that MN rupture is the main mechanism by which DNA becomes cytosolic following ionising radiation exposure and triggering the pathway^3^. Understanding the exact relationship between MN production and ionising radiation could potentially be vital to the development of new treatment planning strategies to exploit natural immunity.

Proton therapy is preferentially used in some cancer types due to its favourable physical dose distribution which delivers very little dose following the range of the protons resulting in a lower integral dose for normal tissue compared to photons. This is therefore suited to treating tumours close to radiation-sensitive structures, reducing dose to healthy tissue distal to the tumour site^10^. In addition, protons have higher linear energy transfer (LET) in the centre and towards the distal end of the Bragg peak, which is associated with more clustered damage around the particle tracks meaning there are more breaks within the localised region which may interact^11–13^. Generally protons have been demonstrated to produce slightly higher levels of damage than traditional photon irradiation, however there are also suggestions that the type of damage may be different. Proton damage may be more complex due to a more focused energy deposition, involving base damage or multiple strand breaks, which are thought to be harder to repair^14^. The combination of these factors may result in a greater cell kill from protons than photons, quantified by the relative biological effectiveness (RBE). RBE is clinically described as 1.1 for protons to represent their slightly higher killing power, however multiple studies demonstrate a variable RBE in vitro and in vivo, specifically changing with LET^15,16^. If irradiation at higher LET results in an increase in DNA damage quantity and complexity, an increase in MN induction may be expected due to greater fragmentation of the DNA.

Previous in vitro studies demonstrate that MN formation is cell line dependent. Guo et al., Slavotinek et al. and Akudugu et al. analysed the MN yield following photon radiation in a combination of cancer cell lines^17–19^. All three studies found that, although there was a strong linear relationship in the number of MN per cell with dose, there was a large difference in the generation of MN across cell lines. This was independent of cell type, species or cancer type. Guo et al. noted an inverse relationship between extent of micronucleation and apoptosis in cell lines^18^. The link between radio-sensitivity and MN yield also remains controversial; Slavotinek et al. demonstrated an increased radio-sensitivity was linked with a higher proportion of MN^19^, however other studies have contrasting results^18,20^.

Several previous papers reported the effect of radiation type and LET on MN induction, with most showing a positive correlation between number of cells containing MN and LET. Sun et al. demonstrated a larger increase in MN production following 18 keV/µm carbon ions and Staaf et al. illustrated a greater MN yield following 97 keV/µm alpha particles^21,22^. However, these MN formation as a function of radiation modality studies only used a single cell type, and the particles used have a much higher LET range than protons. More subtle differences between particle and photon data and lower LET ranges have not been specifically studied. A meta-analysis of studies published containing MN data revealed a large difference in the number of MN generated per dose between and within cell types. Thus, although there is a large amount of published data on MN, it should not be used to study the RBE of different radiation types due to the biological variability in the data. The major contributor to this variation was mainly attributed differences in experimental methods^23^.

To better quantify the relationship between ionising radiation quality, cell line and MN, we studied MN formation in seven cell lines of different cell types following irradiation with photons and protons. To determine any relationship with LET, two proton LETs were used, 0.6 and 6.5 keV/µm (track averaged LET). We demonstrated a linear relationship between dose and the percentage of micronucleated cells for all cell lines and radiation types. Responses between cell lines varied greatly and did not cluster for cell type or p53 status. Trends in response to radiation type were seen for some cell lines, but these were not statistically significant, and the correlation between MN production per dose and LET was not consistent or quantifiable.

**Table 1:** Cell line Characteristics.

Cell Line	Description	Cancerous?	Cell Type (grouped)	P53 Status^*^
RPE1	Retinal Epithelial Tissue	Normal	Head and Neck	Wild Type
RPE1 p53^(−/−)^	Retinal Epithelial Tissue	Genetically Modified Normal	Head and Neck	NULL
H358	Non Small-Cell Lung Cancer	Yes	Lung	NULL
HT1080	Fibrosarcoma	Yes	Muscle	Mutant
RD	Embryonal Rhabdomyosarcoma	Yes	Muscle	Mutant
MG63	Osteosarcoma	Yes	Bone	Wild Type
U2OS	Osteosarcoma	Yes	Bone	Wild Type

*p53 status was extracted from Cellosaurus database^24^.

## Method

### Cell culture

RPE1, H358, HT1080, MG63, RD, and U2OS cell lines, Table 1, were sourced from research groups at the Manchester Cancer Research Centre. All cell lines were authenticated by the Cancer Research UK Manchester Institute Molecular Biology Core Facility. The facility uses the Verogen ForenSeq MainstAY kit for short tandem read (STR) profiling, looking specifically at 27 autosomal STR and 25 Y chromosome STR markers. They compare test results to ATCC references and an internal database of previously tested samples from within the institute. RPE1 p53^(−/−)^ knock out (KO) cells were gifted from Prof Karen Vousden at the Francis Crick Institute, cells were edited as described by Muller et al.^25^. RPE1 and RPE1 p53^(−/−)^ cells were cultured in DMEM-F12 HEPES buffered, supplemented with 2 mM v/v glutamine (Gibco, 11330032). All other cells were cultured in RPMI 1640 media with 2mM v/v glutamine (Gibco, 21875034). All media was supplemented with 10% v/v fetal bovine serum (Gibco, 10270106) and 1% v/v penicillin/ streptomycin (Sigma Aldrich, P0781). Cells were expanded to at least two passages before each experiment and were not passaged more than 25 times accounting for the previous passage count when the samples were acquired.

### Irradiation

Cells were seeded at 750–2000 cells/well into black optical bottomed 96 well plates (Thermo Scientific, 165305) in 200 µl complete media. Plates were irradiated with 2–4 Gy of photon or proton irradiation 32–40 h after seeding. Photon irradiation was performed using a CIX3 irradiator (Xstrahl Inc.) at 300 kV, 10 mA, source-to-surface distance of 400 mm and 2.3 mm thick Cu filter. The time required to irradiate samples was manually inputted based on a measured dose rate of 2.07 Gy/min.

**Fig. 1:**
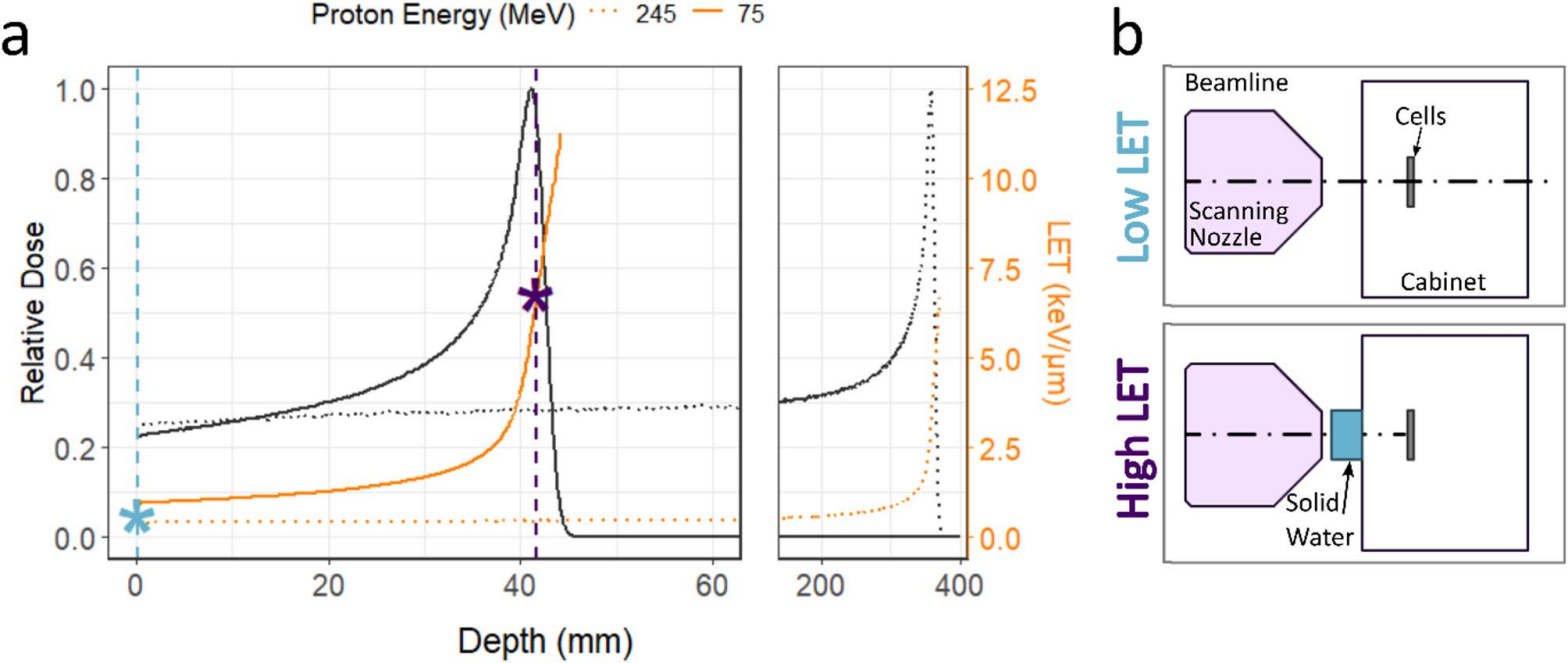
Physical experimental design for protons. (**a**) Single energy (pristine) Bragg peaks created using a 75 MeV beam (solid lines) and a 245 MeV beam (dotted lines). The dose distributions of the beams are shown in black with the relevant calculated LET in orange generated in Geant4 v10.7. Cells were placed at the blue (low LET) and purple (high LET) stars using the experimental setup shown in (**b**). The diagram shows the end of the beam line finishing with the scanning nozzle. Solid water was placed at the end of the nozzle in the high LET setup, outside the radiobiology cabinet to leave enough space in the cabinet for robotic sample handling.

For proton irradiation, two different set ups were created to mimic entry protons and protons at the distal edge of a spread-out Bragg peak (SOBP). The two setups are henceforth referred to as low LET, describing the entry dose protons, and high LET, referring to the distal edge protons. Low LET protons used a single energy 245 MeV beam with a nozzle current of 1.29 nA to produce a track averaged LET of 0.6 keV/µm. High LET protons used a single energy 75 MeV beam with a nozzle current of 0.06 nA and 4 cm of solid water to further degrade the beam to 11.3 MeV on target (calculated through Geant4 Monte Carlo simulation), producing a track averaged LET of 6.5 keV/µm. Figure 1 demonstrates both proton setups with the pristine Bragg peaks from the two energies and their corresponding LET in **a** and the physical placement of the solid water in front of the irradiation cabinet for the high LET setup shown in **b**. Overall irradiation times for low LET protons and photons were similar (around one minute to irradiate with 2 Gy), however irradiation was slower for high LET protons (around 2–3 min to irradiate with 2 Gy).

Monte Carlo simulations were carried out using Geant4 (version 10.7)^23^ for both the experimental set-up with a cell monolayer in a 96-well plate and the dosimetry set-up with a PTW microDiamond (PTW, Freidburg) in place of the cell layer. Dose and LET was scored and a calibration factor was determined to convert dose measured by the microDiamond to that seen by the cells. Proton irradiations were delivered by spot scanning using a pre-defined spot map with spots spacing of 2.5 mm scanning in a snake pattern. Doses were verified prior to sample irradiation with a PTW microDiamond held in situ. Gafchromic EBT3 film was used to ensure homogenous dose across the whole sample at both setups. Radiation was gated using an ionisation chamber. The dose error was < 3% (standard deviation).

### Cytokinesis-Block micronucleus cytome assay and immuno-staining

**Fig. 2:**
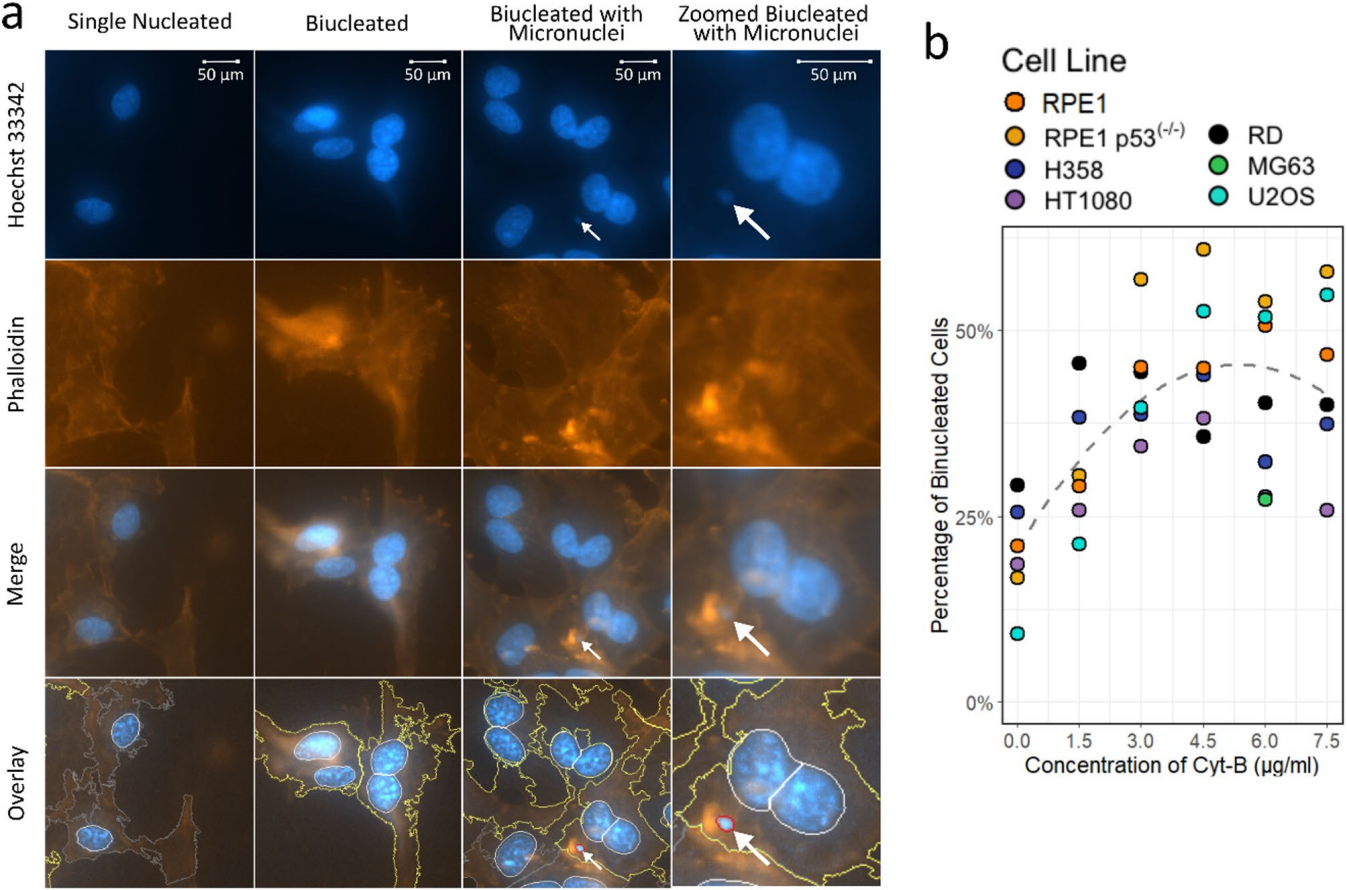
Automation of micronuclei counting and optimisation of cytochalasin-B concentration. (**a**) Immunofluorescence staining of RPE1 p53^(−/−)^ cells and overlay demonstrating the identification of cells in CellProfiler^26^. Single nucleated cells are outlined in grey, binucleated cells are outlined in yellow and the micronuclei (indicated with white arrow) is outlined in pink. (**b**) Optimisation of Cyt-B concentration for the different cell lines. Cells were fixed 24 h after addition of varying concentrations of Cyt-B and the percentage of binucleated cells was calculated using CellProfiler. The grey dashed line represents a polynomial fit to the data calculated in R.

The cytokinesis-block micronucleus cytome (CBMN) assay was performed as described by Fenech and Morley^2^. Cells were incubated at 37ºC for 44 h after irradiation. Cytochalasin-B (Cyt-B) was added to each well in 200 µl complete media at concentration of 4.5 µg/ml. This concentration was optimised for the cell lines using a range of concentrations from 1.5 µg/ml to 7.5 µg/ml, Fig. 2b. There was a general increase in the percentage of binucleated cells up to 4.5 µg/ml as demonstrated by the grey fitted line, however at concentrations above this, the percentage of binucleated cells decreased slightly, particularly in HT1080 and MG63. All cell lines used the same concentration of 4.5 µg/ml, including unirradiated controls. No cell line specific Cyt-B toxicity analysis was performed since results are presented relatively and with control correction.

The plates were then incubated for an additional 24–28 h at 37ºC before being fixed using 10% buffered formalin (Sigma Aldrich, HT501128) and stored at 4ºC. Cells were washed once with phosphate bovine serum (PBS) and permeabilised with 75 µl 0.1% v/v Triton X in PBS (Sigma Aldrich, T8787) for 10 min at room temperature. Plates were then washed once with PBS and 100 µl of a blocking buffer made of 1% w/v bovine serum albumin (BSA) was added for 30 min at room temperature. 50 µl Alexa Fluor 555 Phalloidin diluted in PBS was added to the cells at a concentration of 330 nM v/v and the cells were incubated at room temperature in the dark for 15 min. The plates were then washed three times with PBS and Hoechst 33,342 (Thermo Scientific 62249; 1:1000 diluted in PBS) was added to the cells and incubated in the dark for 10 min at room temperature. The cells were washed three times with PBS and stored at 4ºC.

### Imaging and analysis

Plates were imaged using a Perkin Elmer Operetta high content imaging platform (Perkin Elmer) using a focal water lens at x40 objective. Separate grey scale images were then transferred into CellProfiler v.4.2.4^26^ where bi-nucleated cells and MN were identified through the setup of a new analysis pipeline. The CellProfiler pipeline identified cells through the cytoplasm stain and found binucleated cells by grouping nuclei close together or splitting oddly shaped nuclei. For each cell line, the pipeline was adapted to ensure correct identification using 3–5 images, containing 5–30 cells, and then tested on an additional 3–5 images to ensure there was no significant over-counting or undercounting of binucleated cells or MN. If more than 2 individual nuclei were counted within the same cell when the threshold was increased, the cell was not included in the analysis. Overall CellProfiler correctly identified binucleated cells in 80–95% of cases. Examples of CellProfiler identification of single-nucleated cells, binucleated cells and micronuclei can be seen in the overlay images of RPE1 p53^(−/−)^ cells on Fig. 2a. Within each experiment, three wells of each dose were analysed for each cell line and the data was summed. Only binucleated cells were scored for MN and the results are presented as the percentage of binucleated cells which contain one or more MN (PCMN). Experiments were independently repeated at least three times for each radiation setup and this data was plotted with error bars showing the standard error of the mean.

### Statistical analysis

Quantitative data analysis was performed using R v.4.2.1^27^ and figures were produced using the package ggplot2^28^. Linear regression lines shown in the graphs were carried out in R v4.2.1 using raw data rather than averaged figures. A p value less than 0.05 was considered a significant difference and significance tests were carried out in R v 4.2.1 using single and multivariate ANOVAs.

## Results and analysis

### Scoring 160 binucleated cells resulted in a < 20% fluctuation from the true average in the data sets

**Fig. 3:**
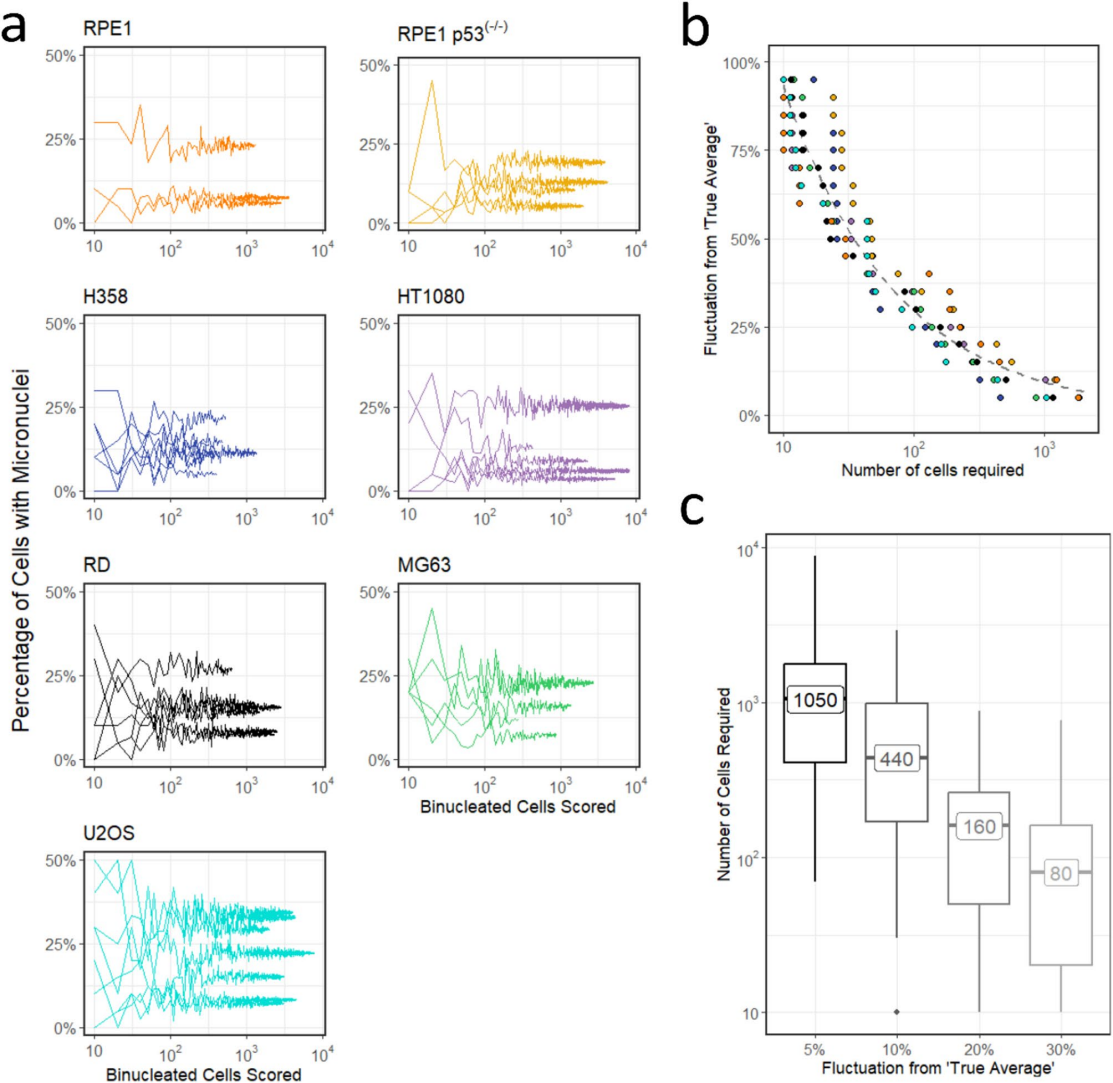
Justification for the number of cells to be scored for each experiment. (**a**) The percentage of cells containing MN vs the number of binucleated cells scored for each cell line. Each line on the plot refers to an individual experiment’s sham dataset. (**b**) These data can be replotted as the fluctuation from the `true average’, which is taken as the value when all cells of the experiment are analysed. The grey dashed line demonstrates the fit of the data which fits the line $\:y=\:\frac{1}{\sqrt{0.115\:x}}$. (**c**) The number of cells required to obtain a fluctuation of less than 30%, 20%, 10% and 5% from the ‘true average’.

For each experiment a non-irradiated (sham) sample was analysed to further understand the fluctuation in the number of MN at baseline. The number of binucleated cells available to be counted ranged from 10 to 7780 cells across the different cell lines. To generate a minimum number of binucleated cells needed for inclusion in the data, random samples were taken of each sham dataset for each cell line. These samples began with a small number of cells and gradually increased in cell number to incorporate the entire data set. The percentage of cells containing micronuclei (PCMN) at baseline was generated for each sample and is plotted in Fig. 3a against the number of cells sampled. Each line on the graph represents one experimental sham for that cell line. These data demonstrate that as more binucleated cells are included from the dataset, the fluctuation in the PCMN at sham decreases and the line tends towards what can be assumed to be the ‘true average’ for this experiment.

Figure 3b plots the fluctuation from this ‘true average’ for each individual experiment and demonstrates that when only 10 binucleated cells are scored, a fluctuation from the true average of up to 100% can be expected. When the number of cells scored is increased above 100, this figure drops to $\:\sim$ 30% and continues to decrease as the number of cells counted increases. As the sampling of the dataset to generate these data fits a binomial distribution, the fluctuation as the number of cells increases can therefore be fitted to an equation of $\:y\:\sim\:\frac{1}{\sqrt{x}}$. These data were found to follow the expected distribution and the fit is demonstrated by the grey dashed line on the plot, following the equation $\:y=\:\frac{1}{\sqrt{0.115\:x}}$. The data are re-plotted in Fig. 3c where the number of cells required for a difference of 30%, 20%, 10% and 5% was calculated. A fluctuation from the true average of 20% was deemed sufficient for this study meaning that at least 160 cells had to be counted for each result. This figure resulted in an error margin of around 2%.

### Variability in the baseline percentage of cells containing MN

**Fig. 4:**
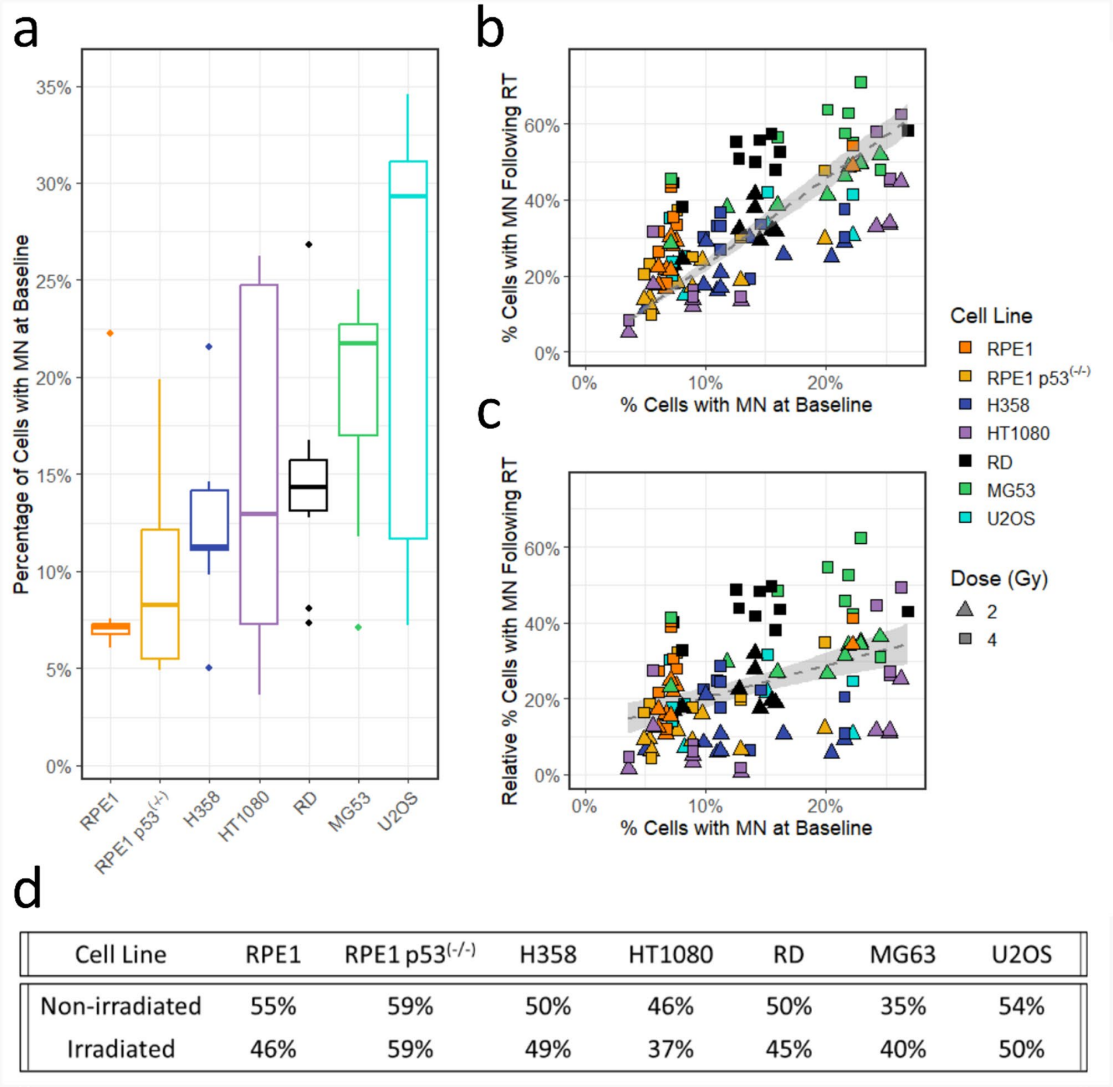
Differences in the sham data sets and the impact on irradiated micronuclei (MN). (**a**) Percentage of cells with MN at baseline for each cell line. (**b**) Effect of baseline MN on the number of cells containing MN following radiation (RT). (**c**) Effect of baseline MN on the number of cells containing MN following RT when the number of cells at baseline is subtracted from the irradiated dataset. Grey lines represent linear regression. (**d**) The percentage of binucleated cells per sample for the non-irradiated and irradiated data sets.

Each cell line had a variation in the number of MN in the sham data across the experiments. U2OS and HT1080 had the largest variation in percentage of cells containing MN before irradiation, whilst RPE1 had the least (Fig. 4a). The number of cells containing MN at baseline was compared to the percentage following irradiation for each individual experiment. A significant, cell line independent, correlation was found, demonstrating that cells which had a propensity for MN induction at baseline, were more likely to contain MN following irradiation (Fig. 4b, ANOVA, F = 245, *p* < 0.001). The data in Fig. 4c demonstrates that this correlation was reduced when the PCMN at baseline is deducted from the percentage following irradiation for each individual experiment. However, there is still a slight increase in the relative percentage of cells containing MN when the baseline dataset contains more MN (ANOVA, F = 85.3, *p* < 0.001). Finally, there was no difference in the number of binucleated cells per sample in the sham and irradiated datasets, Fig. 4d (ANOVA, F = 0.674, *p* = 0.429).

### The impact of radiation on the percentage of cells containing micronuclei

**Fig. 5:**
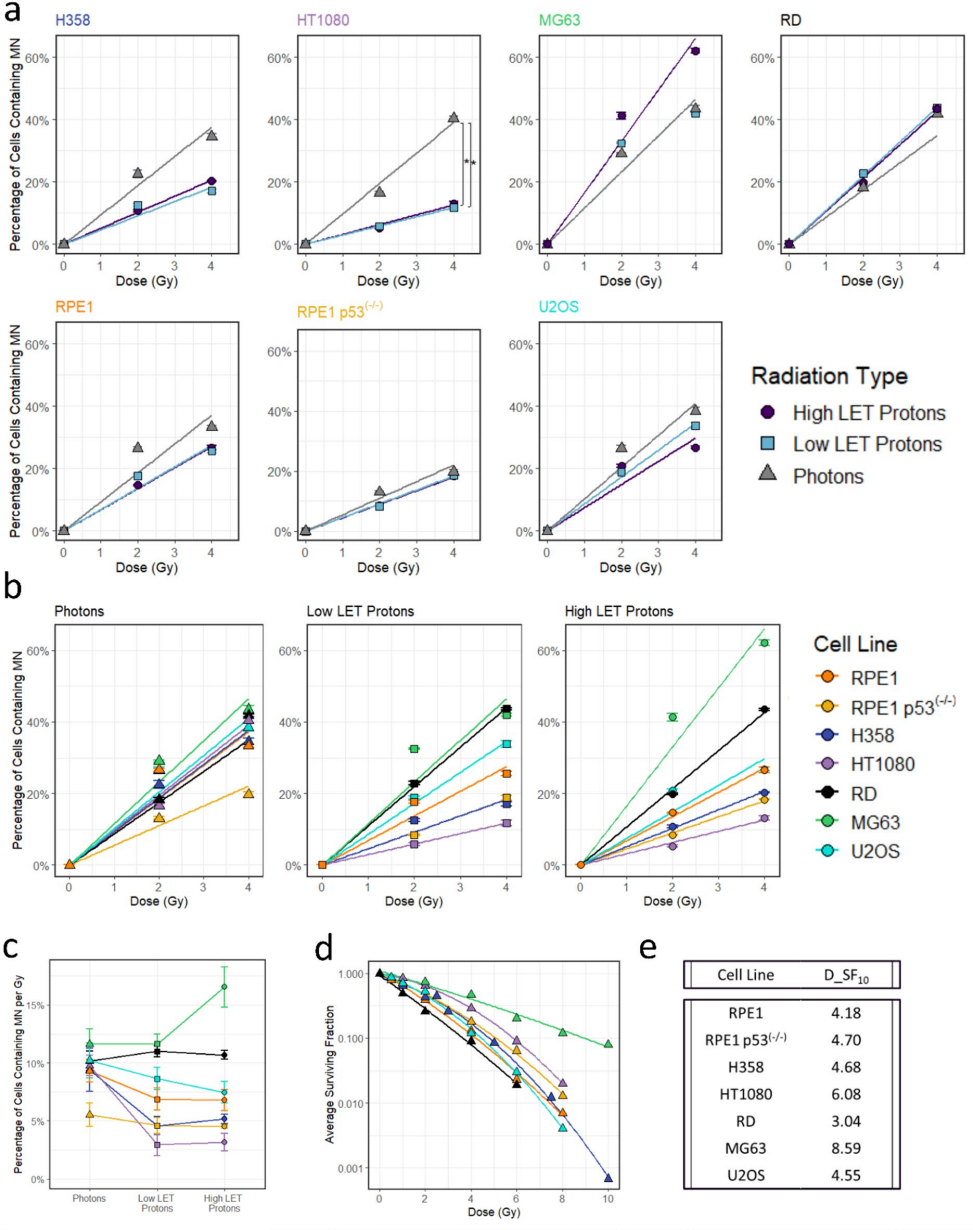
Impact of radiation on micronuclei induction (**a** and **b**) The percentage of cells containing MN following photon, low LET and high LET radiation split by cell type (**a**) and radiation type (**b**). Lines represent the linear regression, weighted by the biological number of repeats. Error bars are the standard error of the mean. (**c**) Percentage of MN per Gy calculated using the slope of the linear regression line across the different radiation types. Error bars represent the standard error of the regression. (**d**) Radiosensitivity of the cell lines to photon radiation from literature. Linear quadratic lines are fitted to the data and the dose (Gy) required for 10% survival (D_SF_10_) calculated (**e**). RPE1 and RPE1 p53^(−/−)^ data was sourced from Guerra Liberal et al.^26^, H358 from Carmichael et al.^29^, HT1080 and RD from Bernardo et al.^30^, MG63 from Zhu et al.^31^ and U2OS from Guerra Liberal et al.^26^.

For all samples there was a strong relationship between dose and PCMN with 4 Gy producing a significantly higher PCMN than the 2 Gy (ANOVA, F = 48.0, *p* < 0.001) as demonstrated in Fig. 5a. Overall, there is a significant variation in the PCMN following radiation between cell lines (Fig. 5b, ANOVA, F = 15.1, *p* < 0.001), with MG63 producing the most MN for each dose. Across radiation types, cells which produce more MN following photon irradiation are more likely to produce more MN following proton irradiation with the exception of RD cells which produce the second least MN after photon irradiation but the second highest number after low and high LET protons, Fig. 5c.

The impact of LET on MN induction varied between cell lines, with no general trend in the effect of LET on PCMN for all data combined (ANOVA, F = 2.09, *p* = 0.24). For individual cell lines there is no significant difference between the PCMN produced following the different radiation setups, with the exception of HT1080 where more cells are micronucleated following photons than high or low LET protons (ANOVA, F = 7.52, *p* = 0.006). Overall, MG63 and RD demonstrate a slight increase in PCMN as LET increases, and the other cell lines show the opposite response although in most cases, there is little LET dependent response demonstrated.

The radio-sensitivity of the cell lines to photon irradiation was evaluated using data previously published and the p53 status was studied using information from Cellosaurus^24^, Table 1; Fig. 5d. The most radio-resistant of the panel of cell lines are MG63 and HT1080 with a photon dose of 8.59 Gy and 6.08 Gy required to reduce survival to 10% respectively, Fig. 5e. The most radio-sensitive cell line was RD with just 3.04 Gy needed for 10% survival. From these data it can be implied that radio-sensitivity is unlikely to be linked to MN production as, of the two radio-resistant cell lines, MG63 produces the highest PCMN from all radiation types and HT1080 the least (ANOVA, F = 39.2, *p* < 0.001). These data also showed that p53 status is also unlikely to be significant, as the p53^(−/−)^ cell lines, H358 and RPE1 p53^(−/−)^ produced a median amount of MN and the p53 WT cell lines, RPE1, HT1080, MG63 and U2OS produced varying levels of MN.

## Discussion

This work explored the differences in radiation induced MN formation between photons and protons. Overall, a dose dependent increase in PCMN was demonstrated for all cell lines and radiation types, however there was no clear quantitative relationship between the PCMN and LET.

Previous variation in MN response was thought to be related to inter-lab variation and protocol discrepancies^23^ and therefore this study aimed to control for experimental technique whilst varying cell line, dose and LET in order to control confounding factors. To further increase the accuracy of the results, a minimum cell number for scoring was established and any effects of sham micro-nucleation were investigated. Although a minimum number of cells counted is usually defined in previous studies, this can vary greatly between 50 and 1000 cells. Ultimately counting an infinite number of cells would achieve the greatest reduction in variation, however this is not a practical protocol and therefore an objective minimum cell number was established to effectively reduce any extreme variation whilst remaining practical to obtain. These results determined that, across this cell panel, counting a minimum number of 160 cells reduced significant variation whilst still being an achievable dataset.

The PCMN in non-irradiated cells was also investigated to highlight any differences in the cell line response caused by spontaneous MN. The effect of baseline MN on MN following irradiation was strong and there was also a large variation in the percentage of cells containing MN at baseline for each individual cell line. The non-transformed cell line (RPE1) had the lowest number of spontaneous MN and also had the least variation in number of MN at baseline suggesting that transformed cells are more likely to micronucleate without external DNA damaging agents. This study assumed that MN formation was mostly from DNA damage resulting in acentric fragments. However, MN can also form from mitotic errors resulting in centric fragments lost at cell division or chromosomal lagging^4,32^. Cancer cells are particularly associated with chromosomal lagging during cell division with a key hallmark of cancer being genetic instability^33^.This could explain the relatively large numbers of MN observed at baseline in some of the cell lines. Centromere specific probes could be used to investigate this phenomenon, identifying both spontaneous and induced MN from centric and acentric fragments respectively.

When subtracted from the post-irradiation data, the baseline numbers of MN have a lesser effect on the dataset. However following radiation there may be a slightly higher percentage of cells containing MN if the baseline sample contained more MN. This did not affect the results of this study as the effect of radiation modality for each cell line was not compared across cells which were significantly more or less micronucleated at baseline. The standard error between experimental repeats also remains low for all cell lines suggesting that subtracting the baseline data, however variable, can give good reliability to the dataset.

The effect of radiation on MN production confirmed a positive relationship between radiation dose and the percentage of cells containing MN across all cell lines and radiation modalities. This relationship was fitted as linear in the analysis but will reach saturation as the percentage of cells containing MN is limited to 100%. For some samples, there is a possibility that the data may saturate at less than 100% of cells containing MN, which can possibly be seen for RPE1 and MG63. However, without more resolution in the data, there are several forms of fitted curve that may be possible. The linear fit for the data in this dose range is strong, with an average R^2^ of 0.86 This suggests it is suitable to describe the majority of the behaviour across the dose range used for these measurements. To achieve a better mathematical description of the behaviour, future work should be conducted with higher dose resolution. As was seen with previous studies^18–20^, there was a large range in the MN production across the different cells, with very few HT1080 and RPE1 p53^(−/−)^ cells containing MN in comparison to MG63 and RD cells which were more easily micronucleated. There was no evidence that these differences were linked to cell type. Of the two osteosarcoma lines (MG63 and U2OS), MG63 produced significantly more MN from all radiation types. This may be due to other underlying differences between the cell lines such as differences in DNA damage and repair and general genetic instability of the cell lines, although there does not seem to be any clustering by broad cancer types. Recent work has also suggested an influence of mitochondria-derived reactive oxygen species (ROS), which has recently been linked to fewer MN due to MN collapse^34^.

The radio-sensitivity of the cells has been previously studied in reference to MN yield. More radiosensitive cell lines were linked to a higher MN yield in some^19^, but not all studies^18,20^. Photon radio-sensitivity was collected from literature for each of the cell lines and this did not have any correlation with PCMN. The specific radio-response to the proton irradiation was not studied, however given no link between photon sensitivity and MN, this is unlikely to be correlated. Another consideration is the early cell survival response following irradiation which may not be captured through clonogenic survival assays. Differences in cell viability within the first 48 h after irradiation are unlikely to be different in normal cell lines, however in cancer cells, where cell death pathways are often bypassed, a small number of cell divisions may be possible. This may affect the MN yield, and could not be captured in a clonogenic cell survival assay which measures stable survival 1–2 weeks later. Further work could explore the correlation between MN production and immediate cell viability following irradiation to further understand this link.

The differences between cell lines were consistent between radiation modalities, with HT1080 producing the least MN from protons and second least from photons across and MG63 micronucleating the most for all radiation types. There remains very little explanation as to why some cells produce more MN than other cells following radiation and there is also very little known about MN rupture. Hatch and colleagues estimate that around 60% of MN rupture within the first 24 h after formation but have not studied cell line differences^6^. Di Bona et al. have recently found a link between mitochondria and MN through ROS, suggesting ROS act as regulators of micronuclear integrity^34^. Although the CBMN attempts to freeze the cells after MN formation by blocking the second cytokinesis, cells with faster cell cycle times, such as RPE1s which double in under 20 h, may have formed MN and had them rupture before the cells were fixed leading to an undercount in the number of MN induced in these cells. There may be further cell specific differences in MN production, with slower cycling cells remaining in cell cycle checkpoints, particularly after 4 Gy, leading to the binucleated cell count being an undercount of the proportion of cells which will divide. Given the cells blocked are likely to have higher degrees of damage, this could result in a lower PCMN at higher doses. There was no obvious doubling time link in this work, with RPE1 and RPE1 p53^(−/−)^ having the shortest cell cycle overall and H358 the longest^35,36^, however this was not specifically studied and could be further investigated in the future, particularly in association with radiosensitivity differences.

A key finding of this work is that the response of cells to LET is inconsistent, with the majority of cell lines demonstrating no significant difference between the different radiation modalities. This is contrary to previous studies using carbon and alpha particle data, which have found that higher LET radiation produces more MN per dose than lower LET radiation^21,22^. These previous studies are limited to one or two cell lines which may have had strong radio sensitivities to different LET and therefore responses shown could have been cell line specific. Previous research also evaluated much higher LET radiation which may have produced more pronounced responses. In this analysis, a variety of cell lines were investigated, including non-transformed cells, cells with specific mutations and a range of cancer types. No single cell line demonstrated a clear quantifiable LET response in MN production and, overall, there was an inconsistent relationship between MN, dose and LET. This relationship may become more clear when irradiating at greater LETs that are unachievable with protons, such as with carbon therapy^22^.

There is a direct relationship between DNA damage and MN, with more damage creating fragments which become MN and therefore it could be expected that higher LET radiation, which increases double strand breaks, should increase the PCMN produced. Given the strong dose response at all radiation modalities, the link between damage and MN is demonstrated in this analysis, however the slightly more subtle LET response is not seen. This suggests that there is more complexity linking DNA damage and MN production. If there is a quantifiable LET link, this may be influenced by differences in damage and repair inside the cell which is too complex to understand from this data.

This dataset provides a foundation for future work in this area which should explore the more complex relationship between DNA damage and MN to understand this critical step in the downstream responses to radiation. MN formation and rupture remain poorly understood and may provide missing links to explain this data. Finally, investigation into the content of MN between cell types may give vital insights into why some cells more readily form MN and whether all MN formed from radiation damage are composed of acentric fragments.

## Conclusion

In conclusion, the field of radiation induced MN has been well researched, however many unknowns surrounding MN production remain. This work explored a panel of cell lines and demonstrates the effect of dose on MN production across different radiation types. Whilst a clear dose dependent relationship was shown for each radiation type in all cell lines, the link between LET and micronuclei production remains unknown and varied across the cell panel. From this, it was therefore impossible to quantify the effect of LET on MN production, suggesting a more complex relationship between dose, MN and LET.

## Data Availability

The datasets generated and analysed during the current study are available on figshare repository, https://doi.org/10.6084/m9.figshare.28451807.v1.
